# Hedonic Consumption in Times of Stress: Reaping the Emotional Benefits Without the Self-Regulatory Cost

**DOI:** 10.3389/fpsyg.2022.685552

**Published:** 2022-05-23

**Authors:** Anna H. Balleyer, Bob M. Fennis

**Affiliations:** Department of Marketing, University of Groningen, Groningen, Netherlands

**Keywords:** acute stress, hedonic consumption behavior, self-regulation, self-control, color-word Stroop task

## Abstract

Hedonic consumption is pleasant but can interfere with the capacity to self-regulate. In stressful moments, when self-regulation is arguably still important, individuals often indulge in hedonic consumption. In two experiments, we investigate whether hedonic consumption negatively affects self-regulation under moderately stressful conditions and whether selecting hedonic consumption under moderately stressful conditions is driven by high or low self-control. In both studies, participants were randomly exposed to a mental arithmetic task that was either completed under time pressure with performance feedback (moderate stress) or without time pressure and without feedback (no stress). Experiment 1 assigned participants to a hedonic (vs. neutral) consumption task and then measured impulse control *via* a color-word Stroop task. Experiment 2 measured self-control as a second independent variable and recorded hedonic (vs. neutral) consumption. The results show that moderate stress buffered the negative effect that hedonic consumption has on self-regulation under no stress conditions and that high rather than low self-control predicts hedonic over neutral consumption under stress. These findings indicate that hedonic consumption in response to moderate stress may be a strategic choice to reap the pleasure benefit of hedonic consumption while the costs to self-regulation are low.

## Introduction

Self-regulation – the ability to control one’s impulses in a way that facilitates the pursuit of valued long-term goals – is essential to adaptive functioning in many domains of life such as health, academic success and relationships. Self-regulation is involved in navigating threats and challenges in pursuit of valued goals and, in particular, steering clear of distractions during goal pursuit (see Vohs and Baumeister, 2016). For instance, focusing on finishing an essay before a deadline rather than browsing social media or snacking requires the individual to control the impulse to indulge in such hedonic consumption as it can be detrimental to subsequent self-regulation and lead to maladaptive consequences in the long-term. Interestingly, hedonic consumption can be adaptive depending on the circumstances in which it is consumed in. The emotion regulation literature, for instance, shows that coping in a high stress situation with mental disengagement is an efficient strategy for saving cognitive resources (Sheppes and Levin, 2013; Dorman Ilan et al., 2019; for a recent review see Sheppes, 2020), suggesting that disengagement from high stress situations with hedonic consumption can similarly be an act of self-regulation. But how might hedonic consumption affect the subsequent capacity to self-regulate in more common, daily and moderately stressful conditions when subsequent self-regulation is likely to be needed? And what are the individual differences that may predict who is more likely to select hedonic consumption under moderately stressful conditions? To answer these questions, we investigate in two experiments how the exposure of hedonic consumption affects self-regulation under moderately stressful conditions and whether high or low chronic self-control predicts hedonic consumption under moderately stressful conditions.

## Hedonic Consumption and Self-Regulation

Indulging in hedonic consumption such as social media, drinks, snacks, video gaming and TV programs is pleasant but can come at the cost of subsequent self-regulation (e.g., Hull and Slone, 2004; Gabbiadini et al., 2014; Holmgren and Coyne, 2017; Exelmans and Van den Bulck, 2018; Thoumrungroje, 2018; see also Hofmann and Fisher, 2012). Indeed, positive affect elicited by hedonic consumption provides the benefit of improving mood and aids emotional recovery (e.g., Macht and Mueller, 2007; Rieger et al., 2015; Schrieks et al., 2016; Roberts et al., 2017; Cook et al., 2019; Johnshoy et al., 2020) but positive affect also reduces various markers of self-regulation such as impulse control (Phillips et al., 2002; Katzir et al., 2010), persistence (Martin et al., 1993; Ceulemans et al., 2013), and increases distractibility (Dreisbach and Goschke, 2004; Rowe et al., 2007), reliance on biases (Bodenhausen et al., 1994; Park and Banaji, 2000), as well as heuristic processing (Mackie and Worth, 1989; Melton, 1995). Hedonic consumption therefore may yield affective benefits even under stressful conditions but this form of consumption may also lead to detriments in self-regulation – at least under no stress conditions. So how would hedonic consumption then affect subsequent self-regulation under moderately stressful conditions?

## Stress – Its Affective and Cognitive Effects

Stress is typically understood as an aversive state which signals threats in the environment. The stress response elicited by stress is characterized by high arousal and negative affective valence which is geared toward causing the individuals to adapt to the changes in the environment (Nesse et al., 2016). While a high stress level and the consequential high stress response leads to adverse cognitive consequences (for reviews see Sandi, 2013; Shields et al., 2016), literature also finds that moderate stress responses can improve various cognitive outcomes (e.g., Henderson et al., 2012; Plessow et al., 2017; Lempert et al., 2018). While the high stress response elicits a fight, flight or freeze response, the moderate stress response recruits cognitive resources allowing more deliberate action. However, since affective and cognitive effects of the moderate stress response are opposing the effects of hedonic consumption, the otherwise negative effect of hedonic consumption on self-regulation is affected by the stress response.

## Stress, Hedonic Consumption, and Self-Regulation

How would indulging in hedonic consumption under moderately stressful conditions predict self-regulation? While the literature on the affect-cognition-link mostly predicts that positive affect leads to less vigilance, more heuristic processing, and decreased self-regulation, we argue that the conditions under which positive affect is experienced are an important factor to consider. Specifically, the stress response signals arising challenges and therefore interrupts the state of low-level processing that is spurred by hedonic consumption. This is in line with the affect-as-cognitive-feedback hypothesis (Isbell et al., 2013; Huntsinger et al., 2014; Ray and Huntsinger, 2017) which suggests that negative affect, such as stress, feedbacks a mismatch between the current cognitive state and the environmental demands. Hence, the signaling prompts an adaptation of attention and processing so that situational demands can be met. Therefore, we hypothesize that hedonic consumption leads to greater detriments in self-regulation under no stress conditions, while under moderate stress, this effect is buffered by the stress response.

## Hedonic Consumption Under Stress – An Act of High or Low Self-Control?

Self-control is often defined as the capacity to inhibit impulses and, hence, the ability to forego short-term temptations in order to pursue long-term goals, whereas low self-control is associated with pleasure-seeking, less consideration for long-term consequences, difficulties to control impulses and limited deliberation (Tangney et al., 2004; de Ridder et al., 2012). With the disposition to discount long-term consequences and to seek pleasure, low self-control individuals engage more in hedonic consumption in research paradigms where immediate pleasure is pitted against the option to pursue normative long-term goals (Kopetz et al., 2018; Jia et al., 2019b; Vosgerau et al., 2020). Within the self-control literature, hedonic consumption is often used in operationalizations of consumptive self-control (failure) which reflects how low self-control and hedonic consumption are almost inextricably linked in this research tradition.

In the self-control literature, hedonic consumption in response to stressful conditions has been attributed to a lack of self-control resources which are needed to suppress the impulse to indulge in hedonic consumption (e.g., Baumeister et al., 1994; Baumeister, 2002). This notion is based on earlier stress literature that found detrimental effects of stress on persistence, attention and cognitive control (Glass et al., 1969; Leon and Revelle, 1985; Keinan, 1987) which are consistent with the resource model of self-control (Baumeister et al., 1994). Longitudinal research, moreover, shows that daily stress is linked to lower situational self-control (Park et al., 2016) and that the link between stress and low self-control behaviors such as hedonic consumption is mediated by impulsivity (Hamilton et al., 2014). This suggests that continued stress also erodes self-control resources and hence leads to more hedonic consumption. Low self-control, therefore, may predict hedonic consumption under stress as stress reduces the self-control resources which are needed to resist indulging in hedonic consumption.

Considered from a regulatory rather than a resource perspective, selecting hedonic consumption when under stress may also be an act of high self-control driven by self-regulatory motives. Several studies have shown that selecting and using hedonic consumption under stressful conditions may be motivated by emotional regulation (e.g., Tice et al., 2001; Atalay and Meloy, 2011; Dorman Ilan et al., 2019). Mead et al. (2016), moreover, show that pleasure can offset stress if it is sufficiently potent and can benefit subsequent goal pursuit and long-term affective wellbeing – which are also hallmarks of high self-control (Tangney et al., 2004; de Ridder et al., 2012). Higher-grade students, who also tend to be higher in self-control (Mischel et al., 1988; Tangney et al., 2004), mostly avoid hedonic consumption but when they indulge in hedonic consumption they do so more deliberately allowing them to reap greater pleasure from it than lower-grade students (Jia et al., 2019a). Taken together, this research suggests that high self-control individuals may be more motivated by emotional regulation under stressful conditions and may reap greater pleasure from hedonic consumption as they mostly refrain from pleasure pursuits. Based on the latter notion, *high* self-control would predict hedonic consumption under stressful conditions if it is indeed an act of self-control. However, if hedonic consumption under moderately stressful conditions is due to the decreased capacity to control impulses as argued above, *low* self-control would predict hedonic consumption under stressful conditions.

## The Present Research

The goal of the present studies was to clarify how moderate stress affects self-regulation when engaging in hedonic consumption and whether the selection of hedonic consumption is driven by high or low self-control. In two experiments, we directly test our hypotheses by manipulating moderate stress and the exposure to hedonic consumption (Experiment 1) or by recording the selection and extent of hedonic over neutral consumption (Experiment 2). While Experiment 1 records the effect of moderate stress and hedonic consumption on impulse control, Experiment 2 measures whether a high or a low level of self-control drives the selection and consumption of hedonic over neutral video clips under moderate stress. Both studies were approved by an ethics committee and only accessible to participants residing in the United States to ensure comprehension of English instructions.

## Experiment 1

Experiment 1 was conducted online and aimed to assess whether moderate stress buffers the detrimental effect of hedonic consumption on impulse control which is a hallmark of self-regulation. This study featured pre-validated video material (see Samson et al., 2016) that was used to manipulate hedonic and neutral consumption.

### Materials and Methods

#### Design and Participants

This first study used a 2 (consumption type: neutral vs. hedonic) by 2 (stress: no vs. moderate) between-subjects design and measured impulse control using a Stroop color-word test (Stroop, 1935). A total of 442 study participants were recruited at Amazon’s mechanical Turk. The mean age of the sample was 35.9 years (*SD* = 10.1; 39% female).

#### Procedure and Measures

Before providing consent, participants were informed that they would be completing a number of tasks and a set of questions as part of a larger project. The study began with a stress manipulation which we described as a mental effort task. Participants were randomly assigned to either a moderate or a no stress condition. The stress manipulation used a mental arithmetic task that induces a moderate stress response (Dickerson and Kemeny, 2004). The task required participants to solve a series of mathematical operations (e.g., “3 * 12 – 29”) after each of which the participants received feedback indicating whether their response was correct. All participants were exposed to the same sequence of mathematical operations, however, in the moderate stress condition participants solved these mathematical operations under time pressure with feedback indicating whether their response was correct. Subjects in the no stress condition were neither exposed to time pressure nor to feedback on their performance.^1^

Immediately following the stress manipulation, participants were asked to pay close attention while watching the video to answer questions about its content later on in the study. Participants were randomly assigned to one of the two consumption type conditions to watch either a hedonic or a neutral video. Both videos were based on emotion-eliciting video material from the Stanford Affective Library (Samson et al., 2016) that were validated to be neutral or positive in affective valence. The neutral video in our study depicted cyclists, a worker grouting and people conversing on a train; the hedonic video featured a baby making faces, a suckling cat and a mishap during a wedding ceremony. The video exposure in both conditions was set to 1 min and 11 s to ensure that the stress manipulation was minimally interfered with and the video material still could elicit an effect.

Next, participants completed the color-word Stroop task to provide a measure of their impulse control. As the Stroop task is provoking impulsive responses by eliciting highly trained and automated behavior (i.e., reading) that needs to be suppressed in favor of a less trained response (i.e., color naming; Stroop, 1935; MacLeod and Dunbar, 1988), the Stroop task has been widely employed as a behavioral measure of impulse control. Before the participants began the task, they were instructed to complete the task as fast and as accurately as possible by indicating the font of color words or a string of X’s per keystroke. Each of the 84 trials displayed either a color word (“blue,” “green,” “red,” or “black”) or a string of X’s in the center of the screen in one of four font colors (blue, green, red, or black). The task comprised altogether three trial types. In congruent trials, the color word matched the font color (e.g., the word “black” in black font), whereas in incongruent trials the color word did not match the font color (e.g., the word “black” in any font color except black). In neutral trials, the “XXX”s were displayed in one of the four font colors. All trial types and font colors were randomized and balanced across the 84 trials.

As online samples may be less attentive than participants in lab studies due to their less controlled and more distractive environment, we assume that response latencies are less sensitive to cognitive interference. Studies conducted by Kane and Engle (2003), for instance, showed that with diminished attentional capacity response latencies are less sensitive measures of cognitive interference compared to error-based scores. We, therefore, focused our analysis of the Stroop task on error rates. Accordingly, we indexed impulse control with a Stroop interference score that was computed by subtracting the average error rate on the congruent and neutral trials from the error rate on incongruent trials. Hence, a larger index represents greater interference with impulse control as the ability to inhibit the prepotent responses decreases.

To assess the success of our stress manipulation, we asked participants to rate their experience during the stress manipulation retrospectively on the six-item state anxiety scale (STAI-6, Marteau and Bekker, 1992). Example items include: “relaxed” (reversed) and “tense.” We averaged the six state anxiety items along with the additional item “stressed” into a single score. As all seven items were rated on a 4-point scale from *not at all* (1) to *very much* (4), a higher score indicates a more stressful experience (*M* = 2.28, *SD* = 0.75, Cronbach’s α = *0.82*). Then, participants rated their experience of watching the video on a four-item 4-point scale, from *not at all* (1) to *very much* (4). Specifically, they indicated to what extent they perceived the video they watched as “fun” (*M* = 2.72, *SD* = 1.12), “entertaining” (*M* = 2.74, *SD* = 1.09), “positive” (*M* = 2.84, *SD* = 0.98), and “neutral” (*M* = 2.63, *SD* = 1.09). Finally, participants were asked to indicate their age and gender to then be thanked, debriefed and compensated.

### Results

#### Preliminary Analyses

We performed a randomization check with age and gender. We have found neither an association between the conditions and age (both at *F* < 1), nor an uneven distribution of gender across the conditions (both χ*^2^* < 1). Hence, we conclude that our randomization was successful.

The stress manipulation check showed that participants perceived the moderate stress condition as more stressful (*M* = 2.36, *SD* = 0.78) than the no stress condition (*M* = 2.19, *SD* = 0.72; *F*(1, 440) = 5.83, *p* = 0.02). As expected, participants also rated the hedonic video more “fun” (*M* = 3.32, *SD* = 0.81), “positive” (*M* = 3.12, *SD* = 0.80), and “entertaining” (*M* = 3.32, *SD* = 0.78) than the neutral video (respectively, *M* = 2.24, *SD* = 1.10; *F*(1, 440) = 133.1, *p* < 0.001; *M* = 2.61, *SD* = 1.05; *F*(1, 440) = 31.9, *p* < 0.001; *M* = 2.27, *SD* = 1.08; *F*(1, 440) = 129.8, *p* < 0.001). Conversely, the participants rated the hedonic video significantly less “neutral” (*M* = 2.23, *SD* = 1.09) than the neutral video (*M* = 2.95, *SD* = 0.98; *F*(1, 440) = 53.4, *p* < 0.001).

#### Target Analysis

**FIGURE 1:**
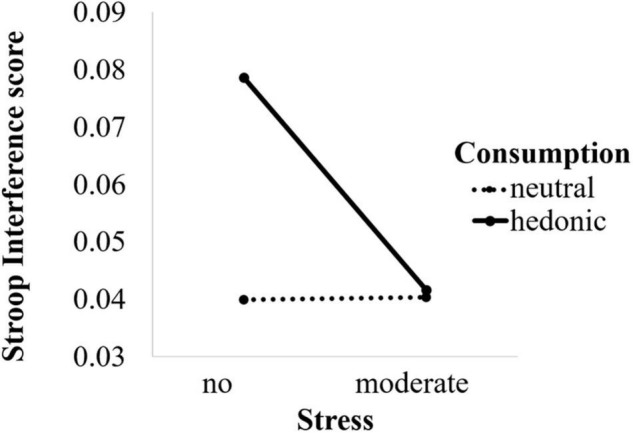
Effects of stress and consumption type on Stroop interference.

To test our notions, we submitted the Stroop interference score to an ANOVA with consumption type and stress as between-subject factors. The results revealed that participants exposed to the hedonic consumption condition showed greater Stroop interference (*M* = 0.06, *SD* = 0.09) than those exposed to the neutral consumption condition (*M* = 0.04, *SD* = 0.08; *F*(1, 438) = 6.70, *p* = 0.01, η^2^ = 0.02). Moreover, participants showed greater Stroop interference in the no stress condition (*M* = 0.06, *SD* = 0.10) than the moderate stress condition (*M* = 0.04, *SD* = 0.07; *F*(1, 438) = 5.77, *p* = 0.02, η^2^ = 0.01). These main effects were qualified by a significant interaction between type of consumption and stress (*F*(1, 438) = 4.33, *p* = 0.04, η^2^ = 0.01). Simple main effect analysis showed that within the no stress condition hedonic consumption (*M* = 0.08, *SD* = 0.11) yielded greater Stroop interference than the neutral consumption condition (*M* = 0.04, *SD* = 0.08; *F*(1, 438) = 11.20, *p* < 0.01, η*^2^* = 0.03, 95% CI_Mean–Difference_ = [0.02, 0.06]). In line with our predictions, moderate stress attenuated the difference in Stroop interference between the hedonic and the neutral consumption condition (respectively, *M* = 0.04, *SD* = 0.06 and *M* = 0.04, *SD* = 0.08; *F* < 1; see Figure 1). Conversely, within the hedonic consumption condition, participants exposed to the no stress condition experienced greater Stroop interference (*M* = 0.08, *SD* = 0.11) compared to participants in the moderate stress condition (*M* = 0.04, *SD* = 0.06; *F*(1, 438) = 9.03, *p* < 0.01, η*^2^* = 0.02, 95% CI_Mean–Difference_ = [–0.06, –0.01]); while within the neutral consumption condition there was no difference in Stroop interference between the no stress and the moderate stress condition (respectively, *M* = 0.04, *SD* = 0.08 and *M* = 0.04, *SD* = 0.08; *F* < 1).

### Discussion

The results show that under conditions of no stress hedonic consumption leads to less efficient impulse control compared to neutral consumption, whereas under moderate stress conditions this performance gap in impulse control is reduced to non-existence. This finding suggests that moderate stress buffers the otherwise detrimental effect of hedonic consumption on impulse control and, hence, self-regulation. As we find that hedonic consumption yields improved impulse control under moderate stress compared to the no stress condition, our findings, therefore, specify the boundary conditions under which hedonic consumption may indeed be a maladaptive choice – that is in the absence of stress.

## Experiment 2

Building on Experiment 1, we used the same stress manipulation and measured rather than manipulated hedonic consumption to assess whether the individuals high or low in self-control select hedonic consumption under moderately stressful conditions. Based on the extant literature, we arrive at two competing hypotheses: First, low self-control may predict hedonic over neutral consumption under moderate stress or, second, high self-control may predict hedonic over neutral consumption under moderate stress conditions. Hence, Experiment 2 tests whether individuals who select hedonic consumption under moderately stressful conditions are indeed individuals who tend to lack self-control or individuals who tend to exert high levels of self-control by measuring trait impulsivity as a proxy for self-control.

### Materials and Methods

#### Design and Participants

This study employed a single-factor (stress: moderate vs. none) between-subjects design with individual differences in self-control as a measured independent variable and with hedonic consumption as dependent variable using video selection and consumption time as its proxies. The sample was recruited at Amazon’s mechanical Turk. After removing three individuals who did not complete the entire survey, the final dataset consisted of 237 participants aged on average 37.5 years (*SD* = 11.8; 44% females).

#### Procedure and Measures

As part of a larger study, participants were told that the online study was made up of several unrelated parts. After providing informed consent, participants completed a stress manipulation identical to Experiment 1. Then hedonic consumption, the key dependent variable in Experiment 2, was measured in two ways: First, we recorded which video participants selected given the choice between a hedonic and a neutral video^2^ and, second, for how long they watched it. We did not provide any specific instructions when presenting the hedonic consumption choice but participants, when presented with the choice between a hedonic and a neutral video, knew from the initial briefing that there were more interview questions to answer about the video and other aspects at the end of the survey. This was to ensure that participants would pay attention to the video as well as to inform them that subsequent task focus was required in order to complete the survey. The hedonic video option titled “compilation of cute animal videos” featured clips of playing or feeding baby animals, whereas the neutral option titled “wildlife documentary” depicted Florida wildlife. Both videos were shortened to last a maximum of 3 min and 11 s in the survey. To reduce bottom and ceiling effects in the consumption time measurement, we computed the difference between participants in consumption time between the hedonic (*M* = 124.0 s, *SD* = 137.0) and the neutral video (*M* = 81.7 s, *SD* = 129.4) to arrive at an overall consumption time score with a higher score indicating longer consumption time of the hedonic over the neutral video (*M* = 42.4 s, *SD* = 236.4).

To assess individual differences in self-control on a low-high continuum, participants were asked to complete the brief Barratt Impulsiveness Scale (Steinberg et al., 2013). Forms of this scale have shown a high negative correlation with trait self-control (e.g., Friese and Hofmann, 2009; Wolff et al., 2016; Mao et al., 2018) and are often used to make inferences about self-control (de Ridder et al., 2012; e.g., Raio et al., 2020). Moreover, the Barratt Impulsiveness Scale proved to be a better predictor for experimental consumption tasks compared to other self-control scales (de Ridder et al., 2012) which we, therefore, consider more suitable to tease out the situational drivers of hedonic motivations in our design. The brief Barratt Impulsiveness Scale used in our design consists of eight items that are rated on 4-point scales from *rarely/never* (1) to *almost always/always* (4). Example items include: “I do things without thinking” or “I plan tasks carefully” (reversed). A higher score, therefore, indicates higher impulsivity. We reversed and averaged the scores to create a single index with a higher score indicating higher self-control (*M* = 3.2, *SD* = 0.5, Cronbach’s α = 0.78).

To assess the success of our manipulation of hedonic and neutral consumption, we asked participants to retrospectively rate the video on four items based on the ten-item Hedonic/Utilitarian Scale (Voss et al., 2003). The four 7-point semantic differential scales were anchored with the bipolar adjective pairs “not fun–fun,” “dull–exciting,” “not delightful–delightful,” and “not thrilling–thrilling” and averaged to create a single index of hedonic perception. A higher score on this index therefore indicates a more hedonic perception of the video (*M* = 4.9, *SD* = 1.2, Cronbach’s α = 0.85). Finally, after providing their age and their gender, participants were debriefed, thanked and compensated for their participation.

### Results

#### Preliminary Analyses

Performing randomization checks on gender and self-control revealed no association between the conditions of our manipulated independent variable (no vs. moderate stress) and the distribution of participants across cells in terms of gender (χ*^2^*(1, 237) = 1.34, *p* = 0.25, *n.s.*) and self-control (*F* < 1), whereas age tended to be associated with stress (*F*(1, 235) = 3.68, *p* = 0.06, *n.s.*). Since age tended to be associated with stress, we controlled for it by including it as a covariate in the target analyses. As expected, we found that the hedonic video was rated significantly more hedonic (*M* = 5.1, *SD* = 1.0) than the neutral video (*M* = 4.7, *SD* = 1.3; *F*(1, 235) = 4.27, *p* = 0.01).

#### Target Analysis

To test our notions, we used the PROCESS macro v 3.2 (Model 1, Hayes, 2018) to run, first, a logistic regression with choice and, second, another regression analysis with consumption time as our dependent variables. In each of the two regression analyses, the dependent variable was regressed on the mean-centered self-control index, dummy-coded stress, and their interaction term. Additionally, both analyses included age as a covariate.

**FIGURE 2:**
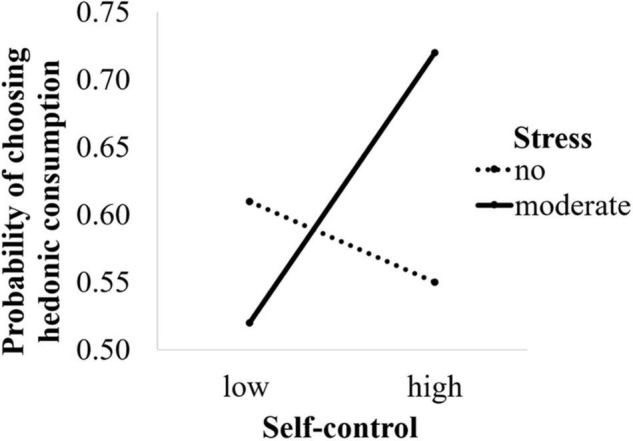
Effects of stress and self-control on the probability of choosing hedonic over neutral consumption.

In the first analysis, we observed non-significant main effects of stress (*B* = 0.17, *SE* = 0.27, *p* = 0.54, *n.s.*), and self-control (*B* = –0.24, *SE* = 0.38, *p* = 0.53, *n.s.*) on choice, however, the interaction between self-control and stress proved to be significant (*B* = 1.08, *SE* = 0.55, *p* = 0.05, *OR* = 0.34). Probing the interaction with a simple slopes analysis, we found that in the moderate stress condition self-control significantly predicted selecting the hedonic over neutral video (*B* = 0.84, *SE* = 0.41, *p* = 0.04), while in the no stress condition self-control did not predict choice (*B* = –0.24, *SE* = 0.38, *p* = 0.53, *n.s*.; see Figure 2) indicating that higher self-control predicted the selection of the hedonic over the neutral video under moderate stress.

**FIGURE 3:**
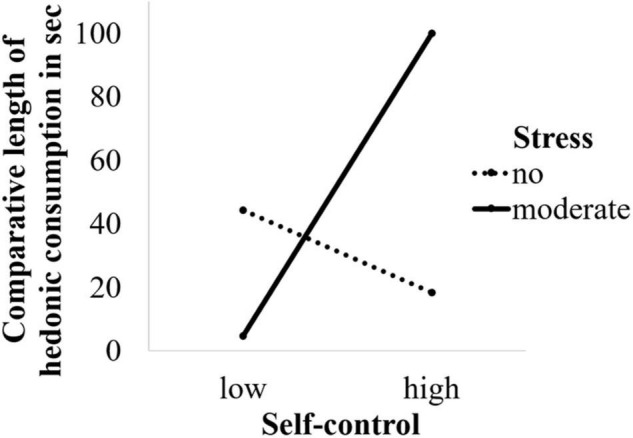
Effects of stress and self-control on comparative viewing time (hedonic video viewing time – neutral video viewing time).

In the second moderation analysis, we observed no significant main effect of stress (*B* = 21.0, *SE* = 30.9, *t* < 1, *n.s.*) or self-control (*B* = –25.8, *SE* = 44.2, *t* < 1, *n.s.*) on consumption time but a significant interaction between stress and self-control (*B* = 120.7, *SE* = 61.5, *t*(232) = –2.0, *p* = 0.05, *r* = 0.16). Additional simple slopes analysis revealed that this interaction paralleled the previous interaction pattern. In the moderate stress condition, self-control had a positive impact on consumption time (*B* = 94.9, *SE* = 44.4, *t*(232) = –2.14, *p* = 0.04), whereas in the no stress condition self-control did not predict consumption time (*B* = –25.8, *SE* = 44.2, *t* < 1, *n.s*.; see Figure 3) indicating that higher self-control predicted longer consumption of the hedonic compared to the neutral video. Removing age as a covariate did not change the pattern of results.

### Discussion

Experiment 2 shows that under moderate stress conditions high self-control individuals are more likely than low self-control individuals to select hedonic over neutral consumption. We find this pattern of results replicated in that high self-control individuals also engage longer in hedonic over neutral consumption under moderate stress conditions compared to low self-control individuals. These findings suggest that selecting and consuming hedonic goods under moderate stress is driven by high self-control and not the lack thereof.

## General Discussion

The present paper explored the consequences and determinants of hedonic consumption under moderate stress to better understand what may drive the phenomenon of selecting hedonic consumption under moderate stress. In two experiments we tested three hypotheses showing that hedonic consumption under moderate stress does not lead to detriments in self-regulation as it does under no stress conditions (Experiment 1) and that the high self-control individuals – not the low self-control individuals – select and engage longer in hedonic consumption under moderate stress (Experiment 2). Therefore, we could not find support for the competing hypothesis that low self-control predicts the selection and consumption of hedonic over neutral videos under stressful conditions. These findings suggest that high self-control individuals strategically reap the benefits of pleasurable hedonic consumption under moderate stress conditions when the otherwise negative effect of hedonic consumption on impulse control is buffered by the moderate stress response.

Hedonic consumption may not always represent a self-control failure as has been outlined by previous research. Hedonic consumption can be undertaken without coming at a cost to goal pursuits as it may be irrelevant to the pertinent goals of the individual (Vosgerau et al., 2020) or even provide benefit to a pertinent goal on a strategic level (e.g., Woolley and Fishbach, study 4; Jia et al., 2019a) and on a tactical level (e.g., Tice et al., 2007).^3^ Specifically in the goal pursuit or self-control context, hedonic consumption has shown to provide affective and motivational benefits to subsequent goal pursuit under no stress conditions (Tice et al., 2007; Coelho do Vale et al., 2016; Prinsen et al., 2018; Jia et al., 2019a) and under stressful conditions (Mead et al., 2016). Extending this research, we investigated the immediate cognitive effects of hedonic consumption under moderately stressful conditions. We furthermore demonstrated that hedonic consumption can be detrimental to the cognitive aspects underlying self-regulation and goal pursuit under no stress conditions but, more importantly, that this negative relationship cannot be generalized to moderately stressful conditions. Under moderately stressful conditions, the detrimental effect of hedonic consumption is buffered by the stress response and, therefore, specifies under which boundary condition hedonic consumption may indeed be (mal)adaptive. Our findings also inform the self-control research. By manipulating the conditions under which hedonic consumption provides a trade-off or not (respectively, under no stress versus moderate stress), we directly pitted the benefitting pleasure aspect of hedonic consumption against the detrimental effect of hedonic consumption on self-regulation. Having found that high self-control individuals robustly select and consume hedonically under moderate stress when hedonic consumption poses no trade-off with self-regulation suggests that high self-control individuals prioritize self-regulatory capacity over pleasure. Meaning that when given the opportunity to indulge without it affecting their self-regulatory capacity, high self-control individuals seize the opportunity to indulge to experience pleasure. This bolsters the argument that individual goals and priorities have to be controlled for when using the consumptive self-control paradigm, as otherwise the interpretation of the choice is rendered arbitrary (e.g., Milyavskaya et al., 2019). Moreover does it suggest that trait high self-control cannot be equated with virtuous choices (see also Uziel and Hefetz, 2014) as this, too, depends on the contexts that high self-control individuals flexibly adjust to and their most pertinent goals at the time of testing.

In Experiment 2, there was no direct test of situational self-control that was underlying the hedonic choice and consumption, rather, we have measured trait self-control as an indirect measure of situational self-control. Although high self-control individual’s selection and consumption of hedonic over neutral videos is robust, these results, however, are merely suggestive of a more self-controlled and deliberate underlying process. Another possible interpretation of our results could therefore be that the selection and consumption of hedonic videos by high self-control individuals under more stressful conditions is not reflective of self-control but rather the lack thereof. In the dieting literature, it is a robust finding that restrained eaters consume more (unhealthy foods) than unrestrained eaters under stress (see reviews by Cardi et al., 2015; Evers et al., 2018). Outside the dieting literature, there is only anecdotal evidence of chronic restraint or self-control leading to less self-controlled behavior under adverse conditions; that is in a study among highly sexually restrained individuals by Nolet et al. (2016) and among high self-control individuals in a study by Imhoff et al. (2014). These studies reason that high self-control individuals may have less practice in making decisions under challenging circumstances as they usually engage in pro-active self-control or avoid temptations more rigorously than low self-control individuals. We recommend that future research takes this into account and tests whether the underlying process may indeed be due to self-control since traits cannot reliably predict behaviors (e.g., Saunders et al., 2018; Milyavskaya et al., 2019).

Both experiments have relied on hedonic and neutral video stimuli to represent hedonic consumption. In both experiments, they have proven to be significantly different in terms of hedonic appeal and were previously validated (study 1). There were several *a priori* reasons to rely on video material as (non)hedonic testing material over other often-used stimuli such as (non)palatable foods or beverages: First, video choice allowed us to use a consequential choice in an online setting and therefore may be less likely to induce demand effects, whereas other types of hedonic consumption remain hypothetical in an online setting and therefore provide less strong evidence. Second, the effects of video stimuli may be less diluted by satiation effects, dietary restrictions or preferences. Video stimuli, therefore, may introduce less noise into the data and, hence, require fewer controls. Lastly, compared to stimuli that have greater sensory appeal, video stimuli may also be interpreted as the more conservative experimental choice from a methodological point of view. We would also assume that testing material with a wider sensory appeal such as the choice between more hedonic and healthy food or beverage options where smell, taste and mouthfeel increase sensory pleasure over and above visual and auditory stimulation would have an even stronger effect on self-regulation. Other research (e.g., Mattila and Wirtz, 2001; Madzharov, 2019) also supports the notion that pleasure derived from hedonic consumption is indeed amplified when it appeals to more senses. However, despite these reasons, there remains a need to examine a wider variety of measures and manipulations of hedonic consumption to strengthen the generalizability of our findings to specific downstream effects that go beyond self-regulatory capacity.

In sum, the present research supports the notion that hedonic consumption may not always be a vice (Kopetz et al., 2018; Jia et al., 2019b; e.g., Ghoniem and Hofmann, 2021), and that stress is not always “bad” (Crum et al., 2020), rather, we specify that under no stress conditions hedonic consumption can be maladaptive, while under moderate stress it may not. Although we have not explicitly measured the effects on long-term self-regulation in our study, other studies have suggested that strategic hedonic consumption can motivate, reward and balance self-control (Coelho do Vale et al., 2016; Mead et al., 2016; Woolley and Fishbach, 2016; Prinsen et al., 2018; Jia et al., 2019a,b). This research indicates that strategic hedonic consumption can help sustain long-term self-regulation and we have demonstrated a possible underlying mechanism that may facilitate this. After all, the occasional strategic hedonic consumption may be just what is needed when working on an essay with a stressful deadline.

## Data Availability Statement

The raw data supporting the conclusions of this article will be made available by the authors, without undue reservation.

## Ethics Statement

The studies involving human participants were reviewed and approved by the University of Groningen. The patients/participants provided their written informed consent to participate in this study.

## Author Contributions

AB conceptualized the study, carried out the experiments, conducted the data analysis, and wrote the manuscript. BF supervised the project, reviewed the manuscript, and provided feedback. Both approved the final version of the manuscript.

## Conflict of Interest

The authors declare that the research was conducted in the absence of any commercial or financial relationships that could be construed as a potential conflict of interest.

## Publisher’s Note

All claims expressed in this article are solely those of the authors and do not necessarily represent those of their affiliated organizations, or those of the publisher, the editors and the reviewers. Any product that may be evaluated in this article, or claim that may be made by its manufacturer, is not guaranteed or endorsed by the publisher.

## Footnotes

1 Exposing participants to a mental arithmetic task was used as a moderate stress induction protocol (cf. Dickerson and Kemeny, 2004; Plessow et al., 2011) as a high stress situation would inhibit any positive effect on performance.

2 The source of the original video material: https://vimeo.com/287521282 (Wilms, 2018) and https://vimeo.com/233520678 (Chaban, 2017).

3 For the analysis of adaptive indulgence on a tactical, strategic, and system level see Jia et al. (2019b).
